# Histone Methylation and Oxidative Stress in Cardiovascular Diseases

**DOI:** 10.1155/2022/6023710

**Published:** 2022-03-16

**Authors:** Xin Yi, Qiu-Xia Zhu, Xing-Liang Wu, Tuan-Tuan Tan, Xue-Jun Jiang

**Affiliations:** ^1^Department of Cardiology, Renmin Hospital of Wuhan University, Wuhan 430060, China; ^2^Cardiovascular Research Institute, Wuhan University, Wuhan 430060, China; ^3^Hubei Key Laboratory of Cardiology, Wuhan 430060, China; ^4^Department of Ultrasound Imaging, Renmin Hospital of Wuhan University, Wuhan 430060, China

## Abstract

Oxidative stress occurs when ROS overproduction overwhelms the elimination ability of antioxidants. Accumulated studies have found that oxidative stress is regulated by histone methylation and plays a critical role in the development and progression of cardiovascular diseases. Targeting the underlying molecular mechanism to alter the interplay of oxidative stress and histone methylation may enable creative and effective therapeutic strategies to be developed against a variety of cardiovascular disorders. Recently, some drugs targeting epigenetic modifiers have been used to treat specific types of cancers. However, the comprehensive signaling pathways bridging oxidative stress and histone methylation need to be deeply explored in the contexts of cardiovascular physiology and pathology before clinical therapies be developed. In the present review, we summarize and update information on the interplay between histone methylation and oxidative stress during the development of cardiovascular diseases such as atherosclerosis, coronary artery disease, pulmonary hypertension, and diabetic macro- and microvascular pathologies.

## 1. Introduction

Cardiovascular diseases, which are mainly considered disorders of the arterial walls, include atherosclerosis, coronary artery disease, pulmonary hypertension (PH), and diabetic macro- and microvascular pathologies. Although numerous advances in diagnosis and treatment have been made, the incidence of cardiovascular diseases is still high. Considering the prevalence of cardiovascular diseases, exploration of the underlying mechanism and development of innovative therapeutic management strategies are urgently needed.

Oxidative stress is defined as the state in which oxidant levels are excessive relative to antioxidant levels in the myocardium and arterial walls under pathological conditions [1–3]. The accumulated reactive oxygen species (ROS), which include superoxide anion radicals (O_2_∙), hydroxyl radicals (OH∙), and hydroperoxide radicals (HO_2_∙), mainly come from mitochondria, NADPH oxidase (NOX), uncoupled nitric oxide synthase (NOS), and xanthine oxidase (XO) in the cardiovascular system [4–6]. However, enzymatic antioxidants such as superoxide dismutase (SOD), catalase (CAT), and glutathione peroxidase (GPx) and nonenzymatic antioxidants such as glutathione (GSH) can remove excess ROS in response to oxidative stress. A large body of evidence has identified oxidative stress as an important pathophysiological mechanism that underlies the initiation, development, and progression of cardiovascular diseases. However, the regulatory mechanism involved in cardiovascular oxidative stress remains poorly understood; therefore, innovative therapeutic strategies to treat and prevent various cardiovascular diseases are lacking.

As numerous studies have elucidated, genetic variations are extensively associated with the initiation and development of cardiovascular diseases. Recently, much influence has also been attributed to environmental and lifestyle factors, such as smoking, aging, and lack of exercise, which modulate epigenetic mechanisms. Epigenetics is the general concept involving the genomic mechanisms that affect gene transcription but without changing the DNA sequence, including DNA methylation, histone modifications (methylation, acetylation, phosphorylation, ubiquitination, adenosine modification, and small ubiquitin modification), long noncoding RNA- (lncRNA-) related mechanisms, and microRNA- (miRNA-) related mechanisms. Among the posttranscriptional modifications, histone methylation is the most well-established and well-studied and is regulated by histone methyltransferases and demethylases. Histone methylation plays several functional roles in the development of cardiovascular diseases. In particular, it regulates oxidative stress by affecting the promoters of related genes that directly encode oxidative/antioxidative enzymes or indirectly exerting important stimulatory effects on cardiovascular oxidative stress signaling pathways. In this review, we provide an overview of the interplay between histone methylation and oxidative stress in the development of cardiovascular diseases.

## 2. Oxidative Stress in Cardiovascular Diseases

Oxidative stress, which is defined as an imbalance between oxidants and antioxidants due to excessive generation of ROS and weak antioxidative defense systems, contributes to oxidative damage to biological components [7–10]. ROS are highly reactive molecules with unpaired electrons that are byproducts of biochemical reactions and primarily include free radicals (e.g., O_2_∙ and OH∙) and nonradical derivatives (e.g., H_2_O_2_) in the cardiovascular system. Reduction of molecular oxygen via transfer of one electron forms O_2_∙, which originates from ROS-producing systems primarily containing enzymes in the mitochondrial respiratory chain, nicotinamide adenine dinucleotide (phosphate) (NADH/NAD(P)H) oxidase, uncoupled NOS, and XO [10–12]. O_2_∙ can be converted to H_2_O_2_ via SOD or via reaction with nitric oxide (NO) to generate highly reactive peroxynitrite (ONOO^−^). H_2_O_2_ can be catalyzed to become water by CAT with or without GPx. GSH provides electrons to GPx. In addition, OH∙ is produced by the reaction of H_2_O_2_ with the reducing transition metal Fe^2+^, a process known as the Fenton reaction.

To detoxify and neutralize accumulated ROS, the cells in heart tissue and vascular walls are equipped with an antioxidant defense system involving antioxidant enzymes, such as SOD, CAT, and GPx, and nonenzymatic antioxidant compounds, such as GSH, to maintain the intracellular redox steady state [13]. ROS formation can be either beneficial or damaging to the cardiovascular system. Under physiological conditions, ROS at low concentrations serve as secondary messengers in signaling pathways that play pivotal roles in cell proliferation and differentiation, transcription factor activation, and gene expression [14–17]. However, excess ROS induce direct and irreversible damage to cellular components such as DNA, proteins, and lipids via oxidative modifications [18–20]. Moreover, excessive O_2_∙ reacts with NO to produce ONOO^−^, which further damages the generation of NO by endothelial NOS (eNOS). Superoxide-induced inactivation of NO leads to endothelial dysfunction. It is well accepted that oxidative stress is related to the initiation and development of a variety of cardiovascular diseases, such as atherosclerosis, myocardial infarction (MI), myocardial ischemia-reperfusion injury (MIRI), PH, and diabetic vasculopathy.

### 2.1. ROS-Generating System in Cardiovascular Disease

Mitochondria are considered the primary sources of endogenous ROS, which are produced via the electron transport chain (ETC) during oxidative phosphorylation. Under normal conditions, nearly 98% of electrons transferred in the ETC are coupled with the generation of ATP, while the rest of the electrons leak out and account for O_2_^−^ production [9, 10]. SOD and CAT are present in mitochondria, and the cytoplasm and can degrade and remove superoxide. Among the four complexes in the ECT, complex I (NADH-ubiquinone oxidoreductase) and complex III (ubiquinone-cytochrome c reductase) are the main generators of O_2_^−^ [21, 22]. Complex I has two sites that generate O_2_^−^. NADH passes electrons to the cofactor FMN in complex I, and then, the reaction of O_2_ with reduced FMN leads to O_2_^−^ production, which is regarded as forward electron transfer [23–25]. The formation of O_2_^−^ at the FMN site is determined by the NADH/NAD^+^ ratio. Reverse electron transfer is another mechanism that generates O_2_^−^ via complex I. When there is a reduced CoQ pool and high *Δ*p, electrons carried by CoQH2 are forced back into complex I, and the Q binding site of complex I is thought to produce superoxide [23, 26]. Complex III is another producer of O_2_^−^. Physiologically, Q sequentially passes electrons through the Fe-S center to cytochrome c. The relatively reactive Q∙ species induced by the transfer of two electrons on CoQ react with oxygen and thus generate O_2_^−^. Typically, the amount of O_2_^−^ generated by complex I is greater than that produced by complex III. Mitochondria account for the majority of cellular ROS production, but they are also significant targets of ROS. Madamanchi and Runge summarized the role of mitochondrial ROS in the development of cardiovascular diseases [27]. Zhong and Kowluru found that mitochondrial damage and dysfunction induced by histone methylation of MMP-9 cause mitochondrial ROS overproduction and thus accelerate the progression of diabetic retinopathy [28]. Other types of mitochondrial ROS are also generated by the inducible redox enzyme p66Shc [29]. After serine-36 phosphorylation by PKC*β*, activated p66^Shc^ moves from the cytosol to the mitochondria and forms a complex with the TIM/TOM mitochondrial import system [30]. When p66^Shc^ is released from the complex, it oxidizes cytochrome c and thus generates H_2_O_2_ [30, 31]. The role of p66^Shc^ in the cardiovascular system has been investigated [32–34]. Inactivation of p66^Shc^ protects against free radical-induced and age-dependent endothelial dysfunction in a mouse model [32]. Moreover, depletion of p66^Shc^ reduces systemic and vascular oxidative stress during the early development of atherosclerosis in mice fed a high-fat diet [33]. However, high p66^Shc^ expression induced by alterations in histone modifications, such as decreased H3K4me2/3 regulated by SUV39H1 and JMJD2C, enhances the progression of ROS-dependent atherosclerosis in obese and overweight mice [34].

Apart from mitochondria, membrane-bound NAD(P)H oxidases are other major sources of intracellular ROS. In contrast to other enzymes that generate ROS as byproducts, NAD(P)H oxidase produces ROS as its the primary function [35]. NADPH oxidase, which is present in cardiovascular system components including endothelial cells, smooth muscle cells (SMCs), cardiac fibroblasts, and cardiac myocytes, and in circulating cells such as monocytes/macrophages, generates O_2_^−^ by transferring electrons from NADPH to oxygen [36, 37]. In mammals, the NOX family consists of seven isoforms (NOX1-5 and dual oxidases 1-2 (DUOX1-2)), which are named according to their core catalytic subunits and regulatory subunits, respectively [38]. Among the seven homologs, NOX1, NOX2, NOX4, and NOX5 are the most commonly expressed in the cardiovascular system [39, 40]. NOX2 was first discovered in endothelial cells and is regarded as the most important ROS generator in vascular disorders. NOX4 plays a controversial role in cardiovascular pathology and generates H_2_O_2_ to protect against cardiovascular disease, as shown by multiple studies [41–43]. Sorescu et al. found that NOX2 and NOX4 are upregulated in atherosclerotic arteries [44]. Similarly, research on endothelial cells subjected to hypoxia-reoxygenation (HR) has shown that KDM3A-induced upregulation of NOX2 and NOX4 contributes to superoxide overproduction in MIRI [45].

NOS has three isoforms (neuronal NOS (nNOS), inducible NOS (iNOS), and eNOS) in humans. eNOS is the main source of ROS under pathological conditions. In the presence of the cofactor tetrahydrobiopterin (BH4) and the substrate L-arginine, eNOS generates NO, which is important for regulating vasodilatation, inhibiting platelet aggregation, and suppressing SMC proliferation [46]. However, eNOS is uncoupled and produces O_2_^−^ under oxidative stress conditions. The reaction of O_2_^−^ with NO produces ONOO^−^, which in turn oxidizes eNOS and BH4 to further produce ROS [47]. Uncoupling of eNOS increases vascular superoxide generation and decreases NO production, and its role in PH has been demonstrated by Zhang et al. [48]. Under pathological situations, iNOS expression is induced in heart tissue and vascular walls [49]. Under conditions of ischemia and hyperglycemia, iNOS is upregulated due to increased H3K4me1/3 modification, which enhances the development of MIRI and diabetic vascular complications [50, 51].

XO is also an important source of ROS in the vasculature. XO catalyzes the conversion of hypoxanthine to xanthine and the oxidation of xanthine to uric acid (the last two steps in purine metabolism). XO provides electrons to molecular oxygen, thus generating O_2_^−^ and H_2_O_2_. Studies have shown that XO increases superoxide production in atherosclerotic lesions [52], while inhibiting XO delays the formation of atherosclerotic plaques in apolipoprotein E- (ApoE-) knockout (KO) mice [53].

### 2.2. Antioxidant Defense System in Cardiovascular Disease

SOD has three different isoforms: SOD1 (copper-zinc-containing SOD or Cu/Zn-SOD), which is mainly located in the cytoplasm and intermembrane space of mitochondria; SOD2 (manganese-containing SOD or Mn-SOD), which is expressed in the mitochondrial matrix; and SOD3 (extracellular SOD or EC-SOD), which is present in the extracellular space. SOD can convert O_2_^−^ into oxygen and H_2_O_2_ and therefore plays a critical role in reducing the vascular oxidative burden. In addition, SOD can maintain NO activation in endothelial cells if superoxide reacts with NO to produce ONOO^−^, which is a highly reactive species with potential cytotoxicity [54]. The three different SOD isoforms are commonly present in the cardiovascular system. SOD1 and SOD2 in the cardiomyocytes and hearts of mice subjected to HR or ischemia-reperfusion (IR) are downregulated by increased H3K9me3 in mice suffering from HR or IR; thus, ROS generation is increased, which accelerates MIRI [55]. Alterations in SOD1 and SOD2 expressions have also been confirmed to occur in MI [56]. In diabetic microvascular complications, SOD2 is essential and related to vascular oxidative stress [57, 58]. Furthermore, downregulation of SOD3 expression modulated by increases in H3K27Ac abrogates the pathogenesis of PH [59].

GPx, which is extensively located in the cytoplasm, nuclei, and mitochondria, can convert H_2_O_2_ into oxygen and water and reduce lipid hydroperoxides to their corresponding alcohols using the tripeptide glutathione GSH as its cofactor. Of all isoforms, GPx1 is the most abundant and is distributed in the cytosol of mammalian cells. In ApoE-KO mice fed a Western-type diet for 24 weeks, GPx1 deficiency promotes the progression of atherosclerotic lesions [60].

CAT also catalyzes the dismutation of H_2_O_2_ to water plus oxygen and is mainly present in the peroxisomes of cardiomyocytes and artery walls. Catalysis depends on the concentration of H_2_O_2_. Generally, CAT functions effectively when H_2_O_2_ is at a high concentration; while in the presence of low concentrations of H_2_O_2_, peroxidase is the main functional enzyme. Numerous studies have revealed that CAT is a critical factor that protects against oxidative stress in cardiovascular diseases. Upregulation of CAT leads to delayed progression of atherogenesis in apolipoprotein-deficient mice [61]. In addition, Zang et al. have found that in cell culture and animal models, CAT knockdown increases total cellular and mitochondrial ROS production and facilitates cardiomyocyte hypertrophy [62].

Heme oxygenase (HO) indirectly decreases ROS production. HO converts prooxidative heme to biliverdin, which can be catalyzed to form bilirubin. Then, bilirubin can scavenge and degrade excessive ROS. In addition, HO modulates the activity of NOX and reduces NOX-derived ROS levels [63]. The role of HO-1 in cardiovascular diseases has been demonstrated. For example, activation of HO-1 by SETD7 exerts a cardioprotective effect against MIRI [64].

The thioredoxin (Trx) system functions as an antioxidant system to detoxify ROS. Its action can be antagonized by the endogenous antioxidant inhibitor Trx-interacting protein (TxnIP) in the vasculature. TxnIP can be stimulated by high glucose due to a carbohydrate response element present in its promoter region. In diabetic nephropathy, EZH2-related downregulation of H3K27me3 represses the transcription of TxnIP to attenuate oxidative injury in podocytes and thus protects against high glucose-induced nephropathy [65].

In addition to enzymatic and nonenzymatic defense systems, the cells have also evolved redox signaling pathways that respond to oxidative stress in the cardiovascular system, including the nuclear erythroid-2-p45-related factor-2 (Nrf2) pathway, the NF-*κ*B pathway, and the Sirt1 pathway. The Nrf2 pathway is very important in clearing and degrading accumulated ROS by regulating the expression of antioxidant defense genes in the vasculature [66–68]. Kelch-like ECH-associated protein 1 (Keap1) binds to Nrf2 and is retained in the cytoplasm, but under oxidative stress conditions, Nrf2 moves into the nucleus to regulate gene transcription [66, 69]. In the setting of high glucose, Nrf2 can be downregulated due to decreased H3K4me1/3. Then, in the nucleus, Nrf2, which is highly sensitive to oxidative stress, upregulates the transcription of related genes, playing an important role in the antioxidant response by binding to antioxidant response element (ARE) 4. ARE4 is an important region in the promoter of glutamylcysteine ligase (GCLC), which encodes a catalytic subunit of glutamate cysteine ligase (Gcl) that is critical for the biosynthesis of GSH to alleviate the progression of diabetic retinopathy [66, 70]. Moreover, the transcription of keap1 is modulated by H3K4me1, and the keap1/Nrf2/ARE pathway also enhances the activation of HO-1 and NADPH-quinone oxidoreductase 1 (NQO1) in diabetic retinopathy [71], which might be the underlying molecular mechanism by which SETD7 protects against MIRI [64].

NF-*κ*B, a redox-sensitive nuclear transcription factor, is highly associated with ROS generation and controls the expression of antioxidant genes in the vasculature [72]. The NF-*κ*B family is composed of five subgroups (RelA or P65, RelB, c-Rel, p50/p105, and p52/p100), members of which are inactive when bound to the inhibitory protein I*κ*B. When stimulated, NF-*κ*B p50/p56 detaches from I*κ*B and translocates into the nucleus to upregulate the expression of related genes. In the context of hyperglycemia, NF-*κ*B stimulation regulated by H3K4 and H3K20 modifications is strongly associated with modulation of the transcription of SOD2 and iNOS, which generates ROS in diabetic retinopathy [50, 58]. In addition, activation of NF-*κ*B modulated by decreased H3K9me2 stimulates the downstream molecule MMP-9, which translocates into mitochondria, damages mitochondrial structure and function, and generates mitochondrial ROS in diabetic microvascular disorders [73]. NF-*κ*B is also activated by oxidative stress to control the expression of many genes in vascular endothelial cells. In human macrovascular endothelial cells, inhibiting the expression and activity of antioxidants such as Mn-SOD or MCP-1 leads to activation of NF-*κ*B p65-induced inflammatory gene transcription, thus aggravating endothelial dysfunction [74].

Silent mating type information regulation 2 homolog 1 (SIRT1) belongs to the sirtuin family and is dependent on the cofactor NAD^+^ for its ability to deacetylate histone or nonhistone substrates to improve transcriptional expression [75]. Much attention has been given to the role of SIRT1 in oxidative stress resistance in cardiovascular diseases. The main pathways include the SIRT1/SOD and SIRT/PGC-1*α* pathways. Transcriptional activation of SIRT1 induced by reduced H3K9me3 upregulates the expression of the antioxidative enzymes SOD1 and SOD2, as evidenced by observations in MI and IR injury, suggesting that SIRT1 exerts cardioprotective effects [55, 56]. Additionally, AMPK cooperates with SIRT1 and activates its common downstream target PGC-1*α*. PGC-1*α*, a key metabolic regulator and sensor, promotes mitochondrial biogenesis to curb oxidative stress by regulating the expression of mitochondrial genes in vascular pathology [76, 77]. Upregulation of SIRT1 medicated by increased H3K79me3 also directly stimulates PGC-1*α* to facilitate the expression of mitochondrial genes and then accelerates the initiation and progression of obesity-induced atherosclerosis [77]. Thus, the SIRT1/AMPK/PGC-1*α* pathway mediates mitochondrial oxidative stress resistance in the vasculature.

## 3. Interplay between Histone Methylation and Oxidative Stress

As numerous studies have elucidated, epigenetic modifications, especially histone modifications, are extensively associated with the initiation and development of cardiovascular diseases. To date, several types of histone modifications have been identified, such as methylation, acetylation, phosphorylation, ubiquitin, adenosine modification, and small ubiquitin modification [78, 79]. However, histone methylation plays an important role in the progression of cardiovascular diseases. Histone methylation is dynamically regulated by histone methyltransferases and demethylases. Methylation of lysine residues in histone N-terminal tails can be catalyzed by histone lysine methyltransferases (HMTs), contributing to the activation or suppression of related gene expression. HMTs, which are mono-, di-, and tri-methylate lysine residues of histones and nonhistones, are divided into two groups: SET domain-containing lysine methyltransferases (KMTs) and non-SET domain KMTs [53, 80]. In contrast, histone demethylases that remove methyl groups from histone tails include Jmjc-domain-containing histone demethylases, which use Fe(II) and *α*-ketoglutarate as cofactors, and lysine-specific demethylases that depend on flavin adenine dinucleotide (FAD) [81, 82]. These methyltransferases and demethylases jointly regulate the methylation of histones and nonhistones. More importantly, methylation at different sites on histones or nonhistones has different biological effects or even diametrically opposed effects. Over the past several decades, numerous studies have demonstrated the involvement of histone methylation and oxidative stress in the pathophysiological processes of a variety of diseases, such as neurodegenerative diseases [83–85], osteoclast-associated diseases [86], intervertebral disc degeneration [87], and cancer [4, 88–92]. A decrease in H3K9me3 is induced by silencing SUV39H1 bound to the SIRT1 promoter, which activates the expression of SOD in MI and MIRI [55, 56]. However, decreased H3K9me2 mediated by KDM3A in the NOX2 and NOX4 promoters promotes NOX2 and NOX4 expression and accelerates the ROS production in MIRI [45]. Similarly, H3K9me2 demethylation mediated by LSD1 repressed MMP-9-modulated mitochondrial ROS production in diabetic retinopathy [28]. These findings collectively indicated that the methylation of histone lysine tails might mainly activate and/or repress gene transcriptions and play important roles in cardiovascular oxidative stress, depending on the site or level of methylation. However, the interplay between the oxidative stress response and histone methylation in cardiovascular diseases has not been sufficiently explored. On the one hand, ROS can modulate histone methylation by affecting the activity and expression of histone methyltransferases and demethylases in the cardiovascular system [93]. For example, the methylation of H3K4 is catalyzed by Set7 binding to the NF-*κ*B p65 promoter in the context of oxidative stress, and this effect is prevented by reducing Mn-SOD-mediated ROS generation in diabetic endothelial dysfunction [74].

Most importantly, histone methylation leads to ROS overproduction in different contexts, such as by activating or repressing the transcription of prooxidant or antioxidant genes by binding to related genes or influencing signaling pathways during the pathogenesis of cardiovascular disorders. For example, H3K9 methylation and binding at the promoters of prooxidants such as SOD cause excessive ROS generation in vascular walls. Moreover, histone methylation affects redox signaling pathways in response to oxidative stress during the development of cardiovascular disorders. Increased H3K4 methylation at the NF-*κ*B promoter stimulates the expression of oxidants such as iNOS to exacerbate macrovascular oxidative stress [50], while increased methylation of H3K4 in the Nrf2 and Keap1 promoters modulates the biosynthesis of GSH in diabetic microvascular pathology [70, 94]. Accordingly, a cycle can form between histone methylation and oxidative stress that plays indispensable and compelling roles in the initiation, development, and treatment of vascular diseases.

In this review, we will clarify the interplay between histone methylation and oxidative stress in cardiovascular diseases such as atherosclerosis, MI, MIRI, diabetic vascular complications, and PH and discuss the potential treatment of these diseases (Table 1).

## 4. Histone Methylation and Oxidative Stress in Cardiovascular Diseases

### 4.1. Atherosclerosis

Atherosclerosis is a chronic inflammatory disorder of the walls of medium and large arteries that is mainly characterized by oxidative stress. ROS generation is increased by several cardiovascular risk factors, such as obesity and aging, which have been verified to accelerate the initiation of atherosclerosis. Obesity increases hypersensitivity to the development of atherosclerosis [95, 96]. Intracellular free radicals originating from mitochondrial p66^Shc^ regulate energetic metabolism and diet-induced obesity [97, 98]. Obesity-induced epigenetic alterations mediated by the methyltransferase SUV39H1, the demethylase JMJD2C, and the acetyltransferase SRC-1 decrease H3K9me2/me3 and increase H3K9 acetylation (H3K9ac), which bind to the p66^Shc^ promoter, causing mitochondrial ROS overproduction in visceral fat arteries (VFAs) from obese subjects compared to nonobese subjects [34]. Consistent with the findings in humans, these epigenetic changes also occur in endothelial cells and in aortas isolated from obese mice. Interestingly, among alterations to the three H3K9-modifying enzymes, the downregulation of SUV39H1 induced by obesity is central to H3K9 modification on the p66^Shc^ promoter, leading to ROS-induced endothelial dysfunction and the development of atherosclerosis [34]. Experiments have verified that the complex of SUV39H1, JMJD2C, and SRC-1 is recruited to the promotor of p66^Shc^ and that JMJD2C and SRC-1 cannot influence the activity of SUV39H1, indicating that targeting SUV39H1 but not JMJD2C or SRC-1 will delay the development of obesity-induced atherogenesis. Age is another dominant risk factor for atherosclerosis. AMPK activation delays aging by reducing mitochondrial oxidative stress. AMPK modulates energy balance and stimulates SIRT1 and PGC-1*α*, which are considered mitochondrial biogenetic markers that play important roles in increasing lifespan by enhancing mitochondrial biogenesis and function. Metformin and AICAR, two AMPK activators, induce H3K79 trimethylation by DOT1L in the promoter of SIRT1/SIRT3, resulting in the expression of telomerase reverse transcriptase (hTERT), which is correlated with enhanced PGC-1*α* expression; this process delays endothelial senescence by enhancing mitochondrial biogenesis/function [99]. Silencing SIRT3 increases mitochondrial oxidative stress and prevents age-induced atherosclerotic plaque formation in ApoE-knockdown mice [99], indicating that hypermethylation of H3K79 via DOT1L leads to SIRT-mediated longevity in the vasculature and that metformin may be a treatment for age-associated atherogenesis.

**Figure 1:**
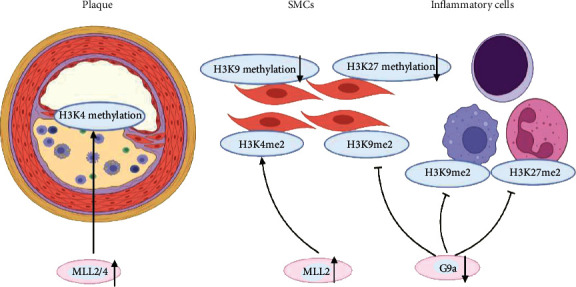
Histone methylation plays an important role in the initiation and progression of atherosclerosis (MLL2: myeloid/lymphoid or mixed-lineage leukemia 2; MLL4: myeloid/lymphoid or mixed-lineage leukemia 4; SMCs: smooth muscle cells).

These findings reveal that risk factors promote the initiation of atherosclerosis by modulating H3K9 and H3K79 methylation and mitochondrial oxidative stress and suggest that targeting SUV39H1 and DOT1L may delay the initiation and development of atherosclerosis. In particular, metformin, which is an important antidiabetic drug, may be beneficial for diabetes-associated atherosclerosis associated with aging. Apart from initiating atherosclerosis, histone methylation can act as a marker of atherosclerosis severity and participate in atherosclerosis progression. In advanced-stage atheroma plaques in human carotid arteries, H3K4me2 is increased in SMCs, H3K9me2 is decreased in SMCs and inflammatory cells, and H3K27me2 is decreased in inflammatory cells in comparison to the levels in early atherosclerosis [100]. The H3K4 methyltransferase MLL2 and the H3K9 methyltransferase G9a are correspondingly upregulated during the advanced stage of atherosclerosis [100]. A recent study has shown that in humans, the levels of H3K9 and H3K27 methylation are decreased in SMCs and inflammatory cells that lack H3K9 methyltransferase expression within advanced atherosclerotic plaques, while H3K4 methylation is increased in SMCs with increased MLL2/4 levels within advanced carotid plaques; these findings indicate that H3K4 methylation might be a marker of atherosclerosis severity [83](Figure 1). The molecular mechanism underlying the formation of atheroma plaques and the progression of atherosclerosis and the interplay between histone methylation and oxidative stress have not been deeply explored and may be novel research directions.

### 4.2. Myocardial Infarction

MI is a major contributor to cardiovascular diseases and is the disorder with the highest mortality and morbidity worldwide. Atherosclerotic plaque disruption with subsequent thrombosis results in occlusion or stenosis of the coronary artery, thus contributing to MI, which is characterized by an inflammatory response, adverse ventricular remodeling, fibrosis, and oxidative stress. Oxidative stress modulated by histone methylation is of great importance in the pathogenesis of MI, and inhibiting ROS generation by regulating histone methylation exerts a cardioprotective effect in MI.

Previous studies have shown that H3K9 methylation participates in cardiac hypertrophy and fibrosis by modulating the generation of radical species [62, 84]. A more recent finding has verified that upregulation of H3K9me2 accelerates adverse ventricular remodeling in MI [85]. Yang et al. found that SUV39H1 deficiency or SUVH39H1 inhibition alleviates cardiac ischemic injury, limits MI size, promotes the survival of mice after MI, reduces cardiomyocyte death, and improves left ventricular function in a SIRT1-dependent manner [56]. Mechanistically, H3K9me3 is methylated by SUV39H1, which is recruited to the SIRT1 promoter. Silencing or inhibiting SUV39H1 with chaetocin attenuates increase in H3K9me3 at the SIRT1 promoter, thus preventing intracellular ROS overproduction [56]. Chaetocin, which is extracted from metabolites of fungal species in the genus Chaetomium, acts as an inhibitor of SUV39H1 and G9a. Recent advances have illustrated that chaetocin exerts various pharmacological effects on cancers, bacteria or viral infections, and cardiovascular diseases by inhibiting apoptosis, oxidative stress, autophagy, and angiogenesis [86], suggesting that chaetocin might have future application value for the treatment of MI by regulating histone methylation and oxidative stress.

### 4.3. Myocardial Ischemia-Reperfusion Injury

Although timely restoration of coronary blood flow via thrombolytic therapy, primary percutaneous coronary intervention (PPCI), and coronary artery bypass grafting (CABG) can limit the infarct size and save the dying myocardium, subsequent acute MIRI can develop and induce further cardiomyocyte death [87–89]. The underlying mechanisms of MIRI are complex and still incompletely understood. Mounting evidence has shown that oxidative stress regulated by histone methylation is a key pathological mechanism in MIRI.

ROS-generating enzymes, including NOX and NOS, are modulated by histone methylation and are involved in the development and treatment of MIRI. The primary function of NOX is to produce ROS, and the NOX2 and NOX4 isoforms are extensively distributed in the myocardium. In response to hypoxia, NOX2 and NOX4 are upregulated by histone methylation or demethylation to induce oxidative stress in MIRI. H3K9 demethylases interact with Brahma-related gene 1 (BRG1), which has the ability to bridge other epigenetic factors to chromatin and change the transcription of target genes to greatly impact MIRI development [90]. For example, KDM3A cooperates with BRG1 to decrease H3K9me2 binding to the promoters of NOX2 and NOX4, thus activating NOX2 and NOX4 expression and inducing ROS overproduction in the context of HR in endothelial cells [45]. Knockdown of Brg1 in vascular endothelial cells reversed oxidative stress by repressing the transcription of the NOX2 and NOX4 genes [45]. NOS is another catalyst for ROS production during MIRI [4], and methylated H3K4 binding at the iNOS promoter contributes to ROS overproduction. Yang et al. observed that myocardin-related transcription factor A (MRTF-A) upregulates iNOS expression in the hearts of mice suffering from MIRI and in cultured macrophages subjected to HR. Mechanistically, MRTF-A coordinates with the H4K16 acetyltransferase TIP60, establishes crosstalk with an H3K4 methyltransferase containing the common subunit ASH2, and increases H3K4me3 in the iNOS promoter, thus inducing the transcription of iNOS and the production of ROS in MIRI [51].

In addition to the oxidant system, the antioxidant defense system and redox signaling pathways (the SIRT1 and NF-*κ*B pathways) have also evolved to protect against the development of MIRI via hypermethylation or hypomethylation of histones. Some investigations have revealed that H3K9 methylation mediates cardioprotective effects against MIRI [91, 92], which is considered the underlying molecular mechanism in ischemic postconditioning (IPC). Demethylation of H3K9 catalyzed by G9a occurs at the promoter of Mtor and alleviates MIRI, reducing the myocardial infarct size and promoting left ventricular function [101]. Several studies have revealed that elevated H3K9me3 levels correlate with SUV39H1 upregulation-induced oxidative stress in I/R hearts and infarcted hearts in vivo and in vitro [55, 56]. SIRT1, a class III protein deacetylase, has been shown to confer cardioprotection against MIRI by curbing oxidative stress [102]. Yang et al. also found that SUV39H1 inhibition or depletion removes H3k9me3 in the promoter of SIRT1, normalizes the expression of SIRT1, and restores SIRT1-mediated upregulation of SOD1 and SOD2 expression, thus reducing oxidative stress and improving cardiac function [55]. In line with the findings related to MI, an inhibitor of SUV39H1, chaetocin, alleviates damage to the myocardium after I/R. Thus, chaetocin is expected to become a potential treatment for both MI and MIRI. Moreover, stimulating the NF-*κ*B/ARE pathway can regulate the expression of HO-1, an antioxidant enzyme that plays a protective role against MIRI. SEDT7 modulates the Keap1/NF-*κ*B/ARE pathway to activate HO-1 and thus alleviates HR-induced injury in cardiomyocytes [64].

**Figure 2:**
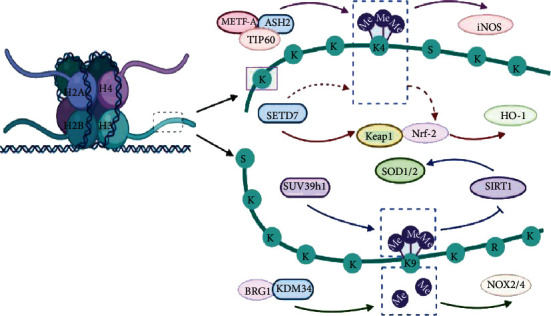
Oxidative stress under the regulation of histone methylation is increasingly being recognized as a target for cardioprotection in MIRI. The upregulation of NOX2/4 and iNOS modulated by KDM3A and ASH, respectively, contributes to oxidative stress in the development of MIRI. The transcription of SOD1/2 and HO-1 is regulated by SUV39h1 and SEDT7, respectively, which accelerates the degradation and removal of ROS in MIRI (ASH: absent small and homeotic; BRG1: Brahma-Related Gene-1; HO-1: heme oxygenase-1; Keap1: Kelch-like ECH-associated protein 1; KDM3A: lysine demethylase 3A; MIRI: myocardial ischemia-reperfusion injury; MRTF-A: myocardin-related transcription factor A; Nrf-2: nuclear factor erythroid 2-related factor 2; NOX2/4: NADPH oxidase 2/4; ROS: reactive oxygen species; SETD7: SET domain containing lysine methyltransferase 7; SIRT1: Sirtuin-1; SOD1/2: superoxide dismutase 1/2; SUV39h1: suppressor of variegation 3-9 homolog 1; TIP60: Tat-interacting protein 60).

As mentioned previously, oxidative stress participates in the development of MIRI by upregulating NOX and NOS transcription or modulating the redox signaling pathway (Figure 2). Targeting histone methyltransferases or demethylases and the redox signaling pathway may be an important therapeutic strategy for MIRI treatment. Additionally, chaetocin is a specific inhibitor of SUV39H1 and may be of great importance. The broad application prospects of chaetocin in the prevention and treatment of MI and MIRI should be further studied.

### 4.4. Pulmonary Hypertension

PH is characterized by a progressive elevation in pulmonary arterial pressure [103]. Mounting evidence has demonstrated that crosstalk between epigenetic modifications and oxidative stress is related to the initiation and progression of PH [48, 59, 104]. NOX4 is the primary ROS-generating enzyme in pulmonary arteries [105]. EC-SOD, which is located in the space between the endothelium and SMCs, not only reduces the reaction of superoxide anions with NO to form ONOO^−^ but also regulates the flow of NO generated from endothelial cells to the smooth muscle layer to stimulate vasodilatation [106]. When human pulmonary artery endothelial cells (HPAECs) and SMCs (HPASMCs) are exposed to the selective histone deacetylase (HDAC) class I and II inhibitor scriptaid and trichostatin A (TSA), acetylation of histone H3 at Lys 27 (H3K27Ac) and H3K4me3 at the gene promoter is increased, which then contributes to increasing the level of EC-SOD and decreasing the expression of NOX4 [104]. In addition to SOD3 (ES-SOD), SOD1 is another isoform that plays a critical role in ROS elimination in the transverse aortic constriction- (TAC-) induced PH models. Inhibition of SOD1 expression by the unregulated H3K27 methyltransferase EZH2 has been observed in a TAC-induced PH model, and the EZH2 inhibitor EPZ005687 has been found to reverse the increase in H3K27me3 at the SOD1 promoter, thus preventing TAC-induced PH [59]. These findings reveal that the selective HDAC class I and II inhibitors scriptaid and TSA and the EZH2 inhibitor EPZ005687 increase SOD expression by influencing H3K4 and H3K27 methylation to attenuate the progression and development of PH, suggesting that these inhibitors may be useful for treating PH patients.

### 4.5. Diabetic Vascular Pathology

Diabetes mellitus and its macro- and/or microvascular complications, including atherosclerosis, nephropathy, and retinopathy, pose a threat to the long-term prognosis of diabetic patients because transient high glucose causes sustained progression of diabetic vascular complications, termed “hyperglycemic memory.” High glucose increases oxidative stress, and increased oxidative stress is considered an important contributor to diabetic vascular complications. Recently, studies have highlighted the crosstalk between histone tail methylation and oxidative stress in diabetes-induced endothelial dysfunction and vascular-related complications [28, 57, 58, 74, 107, 108]. In this section, we clarify the interplay between histone methylation and oxidative stress in diabetic vascular endothelial dysfunction and the progression of diabetic macrovascular and microvascular pathology.

#### 4.5.1. Diabetes-Induced Vascular Endothelial Dysfunction

Vascular endothelial dysfunction underlies the development of macro- and/or microvascular complications resulting from the crosstalk between histone methylation and oxidative stress. NF-*κ*Bp56 bridges the interaction between oxidative stress and histone methylation in the context of diabetes. First, mitochondrial oxidative stress alters NF-*κ*Bp56-induced methylation of histones at related promoters, promoting the inflammatory response in diabetic endothelial dysfunction. For example, transient hyperglycemia persistently mobilizes the histone methyltransferase Set7 to bind with the promoter of the NF-*κ*Bp65 gene in bovine aortic endothelial cells (BAECs) and human aortic endothelial cells (HAECs). The activation of NF-*κ*B in turn induces the transcription of inflammation-associated genes, leading to vascular endothelial permeability and leukocyte adhesion which accelerates endothelial dysfunction. This effect can be abolished by overexpression of either uncoupling protein-1 (UCP-1) or Mn-SOD [74]. Mn-SOD, which is mainly present in mitochondria, primarily functions in discomposing and reducing superoxide. The stability of the mitochondrial redox state regulates the inflammatory response in endothelial dysfunction in the context of hyperglycemia. In addition, histone methylation participates in endothelial dysfunction by methylating lysine residues on histones enriched in the promoter of NF-*κ*B, stimulating the NF-*κ*B signaling pathway to regulate oxidative stress by affecting the expression of prooxidants. For example, H3K4 monomethylated by Set7 regulates the expression of COX-2 and iNOS by binding to the NF-*κ*Bp65 promoter in peripheral blood monocytes (PBMs) from patients with type 2 diabetes mellitus (T2DM), thus inducing ROS and inflammatory factor production [50]. Silencing Set7 blunts NF-*κ*B-dependent oxidant and inflammatory signaling in HAECs cultured in high glucose conditions [50]. As mentioned previously, the NF-*κ*B pathway promotes the formation of a cycle of oxidative stress and histone methylation in vascular endothelial dysfunction in the context of diabetes. Based on the functions of Set7 in diabetes, diabetic vascular complications, cancers, and atherosclerotic vascular disease, inhibition of Set7 might be beneficial for prevention or treatment of diabetes-related diseases. DC21, a newly synthesized selective compound that is a potent inhibitor of Set7, might have significant preclinical or clinical applications for diabetic vascular complications [109].

The expression of NOX and NOS is modulated by histone methylation, and these molecules are involved in vascular endothelial cells in diabetes mellitus. Temporary hyperglycemia persistently upregulates H3K4me1, H3K9me2, and H3K9me3 at the promoters of NOX4 and eNOS, which are sources of vascular ROS production in endothelial cells [110]. Interestingly, one study has shown that increased H3K4me1 is correlated with LSD1 knockdown, while SUV39H1 is considered to play a weak role in regulating H3K9me2 and H3K4me3 at the Nox4 and eNOS promoters [110], suggesting that LSD1 is a key factor in endothelial dysfunction and that targeting LSD1 may be an important method of reducing diabetes-associated endothelial dysfunction. A large number of small molecular LSD1 inhibitors including TCP, ORY-1001, TCP, ORY-2001, and GSK-2879552 have been developed. However, these LSD1 inhibitors have all been applied in clinical trials to prevent cancers [111]. In the future, investigation into whether and how the above LSD1 inhibitors affect diabetic diseases may provide another additional therapies to prevent the progression of diabetic vascular complications.

The above findings reveal that H3K4 and H3K9 methylation both play significant roles in oxidative stress in the dysfunction of endothelial cells subjected to high glucose concentrations via Set7 and LSD1. However, the role of SUV39H1 in hyperglycemia needs to be further investigated.

#### 4.5.2. Diabetic Vascular Complications

Although the epigenetic regulation of diabetic macrovascular complications has been reviewed by some investigators, few studies have examined the crosstalk between histone methylation and oxidative stress in macrovascular complications of diabetes [84]. Atherosclerosis is a common diabetic macrovascular complication, and the NF-*κ*B pathway plays a very important role in modulating oxidative stress. Lipopolysaccharide (LPS) stimulates the NF-*κ*B signaling pathway and thus induces the expression of JMJD3 (KDM6B). When NF-*κ*B/p65 and Jmjd3 bind to the related promoter region, H3K27me3 can be further decreased in the context of oxidative stress and affect wound angiogenesis. Collectively, the evidence indicates that JMJD3 might be a novel target for alleviating the development of atherosclerosis [112].

Among all diabetic microvascular complications, retinopathy and nephropathy are the most common complications and are associated with high rates of disability and mortality. Overproduction of ROS plays a significant role in the development of diabetic microvascular disorders. A series of studies have shown that high glucose alters H3K9 methylation levels in both cells and animals. For example, Yu et al. have illustrated that the levels of H3K9me3 are decreased by SUV39H1 in cardiomyocytes cultured in high glucose [113], which is consistent with the results obtained in SMCs from mice and humans [114]. In addition, animal and human experiments have revealed the decreased levels of H3K9 methylation. Zhong and Kowluru demonstrated that H3K9 demethylation is downregulated in the retina from humans with an average diabetes duration of 10-30 years [28]. Stimulation of the NF-*κ*B pathway activates MMP-9 translocation into mitochondria and induces mitochondrial dysfunction. High glucose decreases the dimethylation of H3K9 regulated by LSD1 and increases the phosphorylation of NF-*κ*B P56 at the MMP-9 promoter, resulting in mitochondrial ROS overproduction in diabetic retinopathy [28]. TxnIP can inhibit the activity of Trx, an important antioxidant that scavenges excess ROS in the vasculature. Siddiqi et al. found that depletion of EZH2 decreases H3K27me3 recruitment to the promoter of the transcription factor Pax6 and upregulates the expression of TxnIP to induce oxidative injury in podocytes under high-glucose conditions [65]. Conversely, the S-adenosylhomocysteine hydrolase inhibitor 3-deazaneplanocin A (DZNep) degrades EZH2 and then upregulates TxnIP and increases oxidative stress in podocytes [65]. Thus, activation or stimulation of EZH2 might be of great therapeutic value. Although few activators of EZH2 have been explored, their development may be promising for the prevention and treatment of diabetic vascular complications in the future.

SOD, a member of the antioxidant defense system, plays a protective role in the pathogeneses of diabetic microvascular complications. Zhong and Kowluru observed that transient hyperglycemia increased H4K20me3, acetylated H3K9, and NF-*κ*B p65 levels at the promoter and enhancer of retinal sod2, which encodes Mn-SOD, reducing mitochondrial overproduction of superoxide and contributing to downregulation of Sod2 expression in animal models and retinal endothelial cells [58]. Two years later, Zhong's team showed that high glucose decreased H3K4me1 and H3K4me2 at the Sod2 promoter in cultured retinal endothelial cells and the retinas of diabetic rats and human donors [57]. Silencing LSD1 attenuated the decrease in H3K4 methylation and reversed the change in the expression of Sod2 [57]. SUV420h2 and LSD1 both repress Sod2 transcription to reduce the ability of Sod2 to remove ROS and thus contribute to oxidative stress during the development of diabetic retinopathy. However, the role of LSD1 has been confirmed in donors with diabetic retinopathy, and its role is different from that of SUV420h2, indicating that targeting LSD1 may be a better potential strategy to slow the progression of diabetic retinopathy than targeting SUV420h2. Biological small molecular inhibitors of LSD1 with important clinical activity, especially ORY-1001, can be applied into the treatment of diabetic vascular complications. Furthermore, a potent, selective, and cell-active inhibitor of SUV420h2, A-196, has been discovered. A-196 selectively inhibits the activity of SUV420h2 and might prevent and alleviate the progression of diabetic vascular complications [115].

**Figure 3:**
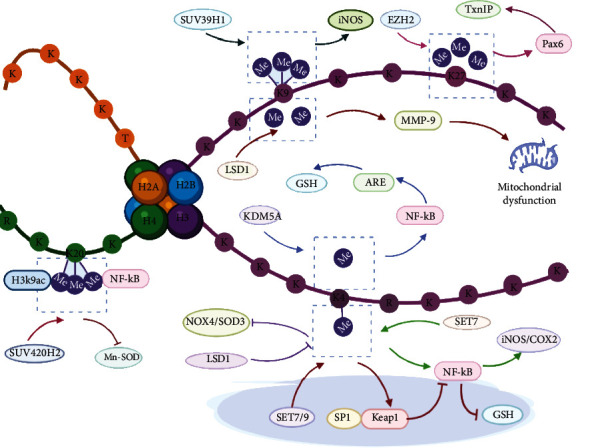
Histone methylation modulates oxidative stress in diabetic endothelial dysfunction and vascular complications. High levels of MMP-9 and TxnIP due to upregulation by LSD1 and EZH2 increase ROS overproduction in diabetic microvascular pathology. The expression and activity of Mn-SOD are decreased by both SUV420h2 and LSD1 in diabetic retinopathy. The Nrf2/Keap1 signaling pathway eliminates ROS in diabetic patients with retinopathy. KDM5A and SET7/9 both modulate the Nrf2/Keap1 signaling pathway and thus influence GSH biosynthesis (COX2: cyclooxygenase-2; EZH2: enhancer of zeste homolog 2; GSH: glutathione; Keap1: Kelch-like ECH-associated protein 1; KDM5A: lysine demethylase 5A; LSD1: lysine-specific demethylase 1; MMP-9: matrix metalloproteinase-9; NOX4: NADPH oxidase 4; ROS: reactive oxygen species; SET7/9: (su(var)-3-9,enhancer-of-zeste,trithorax) domain-containing protein 7/9; SOD3: superoxide dismutase 3; SP-1: specificity protein-1; SUV39H1: suppressor of variegation 3-9 homolog 1; SUV420H2: suppressor of variegation 4-20 homolog 2; TxnIP: thioredoxin-interacting protein).

In addition to antioxidative enzymes, the antioxidant Nrf2/Keap1 pathway also eliminates ROS in diabetic patients with retinopathy. Epigenetic modification at the promoters of Keap1 and Nrf2 modulates oxidative stress during the progression and development of diabetic retinopathy [70, 94]. Keap1 prevents Nrf2 translocation from the cytosol to the nucleus to bind with ARE4, thereby downregulating the transcription of GCLC and largely attenuating the biosynthesis of GSH, indicating that the Nrf2-Keap1-GCLC pathway plays an important role in diabetic retinopathy [28]. Hyperglycemia impairs Nrf2 binding at Gclc-ARE4 due to increased H3K4me2 and decreased H3K4me1/me3 in retinal endothelial cells and the retinas of rats and human donors with diabetes, and silencing LSD1 ameliorates the decrease in H3K4me1 and prevents the biosynthesis of GSH in diabetic retinopathy [70]. These findings suggest that LSD1 inhibitors may be important treatment agents for diabetic retinopathy. In addition to H3K4me2 in the Nrf2 promoter, the methylation of H3K4 by both Set7/9 and stimulated protein-1 (SP1) is enriched in the Keap1 promoter, which promotes the biosynthesis of GSH and regulates oxidative stress in diabetic retinopathy [94]. Recent findings have revealed that a specific inhibitor of Set7/9, cyproheptadine, alleviates the development of renal ischemia injury in diabetic and nondiabetic rats [116], indicating that cyproheptadine might be a novel therapeutic agent for diabetic retinopathy. Hyperglycemia induces the Nrf2/Keap1 pathway and the NF-*κ*B pathway to stimulate the oxidative stress response. Histone posttranscriptional modifiers such as histone methyltransferases and demethylases that catalyze the methylation of histone tails on promoters affect these signaling pathways, inducing oxidative stress in the context of diabetic vascular complications, especially microvascular complications (Figure 3).

## 5. Conclusion and Perspective

Cardiovascular diseases are the leading cause of sudden death and pose great burden worldwide. Genetics is involved in the development of cardiovascular diseases such as atherosclerosis, coronary artery disease, PH, and diabetic macro- and microvascular pathologies. Epigenetic modifications regulated by other factors such as environmental and lifestyle factors greatly influence the progression of cardiovascular diseases. In addition, oxidative stress is a consequence of ROS overproduction that overwhelms the elimination ability of antioxidants and plays a significant role in cardiovascular diseases. ROS-generating components, including mitochondria, NOX, NOS, and XO, create superoxide overload in cardiovascular diseases. The myocardium is equipped with an antioxidant system involving enzymes and nonenzymatic compounds such as SOD, CAT, Prx, HO, and Trx and GSH to neutralize and detoxify excess ROS. Redox signaling pathways, primarily the Nrf2 pathway, NF-*κ*B pathway, and Sirt1 pathway, are also involved in cardiovascular diseases. As we have discussed, oxidative stress regulated by histone methylation plays a critical role in the development and progression of cardiovascular diseases by affecting the expression of oxidative/antioxidative enzymes or modifying redox signaling pathways. Targeting the underlying molecular mechanism to alter the interplay of oxidative stress and histone methylation may enable the development of creative and effective therapeutic strategies against a variety of cardiovascular disorders. Recently, some drugs targeting epigenetic modifiers have been used to treat specific types of cancers. However, the comprehensive signaling pathways that bridge oxidative stress and histone methylation in cardiovascular physiology and pathology need to be thoroughly explored before clinical therapies be developed. Furthermore, the existing evidence that targeting the methylation and demethylation of histones is beneficial for cardiovascular disorders has largely comes from cellular and animal experiments.

## Figures and Tables

**Table 1:** Histone methylation and oxidative stress involved in the development and progression of cardiovascular diseases.

Types of CVDs	Modifying enzymes	Changes of histone methylation levels	Effects on oxidative stress
Atherosclerosis	SUV39H1	Decreased H3K9me2/3 (human and mice)	Increased P66shc and oxidative stress [34]
	DOT1T	Increased H3K79me3 (mice)	Stimulated AMPK/SIRT/PGC1 and increased oxidative stress [99]
	MLL2 and G9a	Increased H3K4me2 in SMCs, decreased H3K9me2 in SMCs and inflammatory cells, and decreased H3K27me2 in inflammatory cells (human) [100]	—
	MLL2/4	Decreased H3K9 and H3K27 methylation in advanced atherosclerotic plaques of SMCs and inflammatory cells (human) [83]	—
MI	SUV39H1	Increased H3K9me3 (mice)	Downregulated SIRT and upregulated SOD1/2 [56]
MIRI	KDM3A	Decreased H3K9me2 (mice and human endothelial cells)	Increased NOX2/4 and oxidative stress [45]
	ASH2	Increased H3K4me3 (mice)	Increased iNOS [51]
	G9a	Increased H3K9me2 (mice)	Regulating cardiac autophagy [101]
	SUV39H1	Increased H3K9me3 (mice)	Downregulated SIRT and upregulated SOD1/2 [55]
	SEDT7	Increased H3K4me1 (cell)	Stimulated Keap-1/Nrf-2 pathway [64]
PH	—	Increased H3K27Ac and H3K4me3 (cell)	Increased EC-SOD and decreased NOX4 [104]
	EZH2	Decreased H3K27me3 (mice)	Increased SOD1 [59]
Diabetic vascular complications	Set7	Increased H3K4me1 (human and mice)	Increased COX-2 and iNOS [50]
	LSD1	Decreased H3K4me1 (mice)	Increased Nox4 [110]
	SUV39H1	Increased H3K9me2/3 (mice)	Increased eNOS [110]
	SUV39H1	Decreased H3K9me3 (cell)	Increased IL-6, MCSF, and MCP-1 expressions [113]
	SUV39H1	Decreased H3K9me3 (cell)	Increased IL-6 expression [114]
	LSD1	Decreased H3K9me2 (cell, mice, and human)	Stimulated NF-*κ*B P56 and upregulated MMP-9 [28]
	EZH2	Decreased H3K27me3 (mice)	Increased TxnIP [65]
	SUV420h2	Increased H4K20me3 (cell and mice)	Decreased Mn-SOD [58]
	LSD1	Decreased H3K4me1/2 (cell, mice, and human)	Decreased Mn-SOD [57]
	KDM5A	Decreased H3K4me1/3 (cell, mice, and human)	Inhibited Nrf2/ARE4 and reduced GSH [70]
	Set7/9	Increased H3K4me1 (cell, mice, and human)	Increased Keap1 and inhibited Nrf2/ARE4 and reduced GSH [94]

ASH2: absent small and homeotic 2; COX2: cyclooxygenase-2; CVDs: cardiovascular diseases; DOT1L: disruptor of telomeric silencing 1-like; EZH2: enhancer of zeste homolog 2; GSH: glutathione; Keap1: kelch-like ECH-associated protein 1; KDM3A: lysine demethylase 3A; KDM5A: lysine demethylase 5A; LSD1: lysine-specific demethylase 1; MI: myocardial infarction; MIRI: myocardial ischemia-reperfusion injury; MMP-9: matrix metalloproteinase-9; NOX2/4: NADPH oxidase 2/4; PH: pulmonary hypertension; SETD7: SET domain containing lysine methyltransferase 7; SET7/9: (su(var)-3-9,enhancer-of-zeste,trithorax) domain-containing protein 7/9; SOD1/2: superoxide dismutase 1/2; SUV39H1: suppressor of variegation 3-9 homolog 1; SUV420H2: suppressor of variegation 4-20 homolog 2; TxnIP: thioredoxin-interacting protein.
